# An FSM-Assisted High-Accuracy Autonomous Magnetic Compensation Optimization Method for Dual-Channel SERF Magnetometers Used in Weak Biomagnetic Signal Measurement

**DOI:** 10.3390/s25123690

**Published:** 2025-06-12

**Authors:** Xinran Tian, Bo Bao, Ridong Wang, Dachao Li

**Affiliations:** State Key Laboratory of Precision Measurement Technology and Instruments, Tianjin University, Tianjin 300072, China; tiantxr0607@tju.edu.cn (X.T.); baobo@tju.edu.cn (B.B.); dchli@tju.edu.cn (D.L.)

**Keywords:** SERF atomic magnetometer, magnetic field compensation, finite state machine, iterative optimization, biomagnetic measurement

## Abstract

**Highlights:**

**What are the main findings?**

- FSM-IOMCA Algorithm: A novel finite state machine-assisted iterative optimization method achieves pT-level compensation resolution (error < 1.6%) and 38% higher sensitivity compared to the IHCA algorithm.
- Single-Beam Dual-Channel Design: Utilizes a 1 × 2 polarization-maintaining fiber (PMF) for a miniaturized SERF magnetometer and establishes a single-beam dual-channel system.

**What is the implication of the main findings?**

- Stability and high accuracy: The FSM framework ensures robustness and enables the automation of magnetic compensation; The iterative optimization method improves the accuracy, reduces the crosstalk between axes and probes, enhances the performance of biomagnetic measurement system, and makes the detection of weak biomagnetic signals more accurate and reliable.
- Array-based biomagnetic sensing potential: 1 × 2 PMF can be extended to 1 × N PMF to achieve multi-channel biomagnetic measurement, which is crucial for biomedical applications (e.g., magnetocardiography, magnetoencephalography, and biomarker detection of magnetic markers).

**Abstract:**

Atomic magnetometers based on the spin-exchange relaxation-free (SERF) regime have broad applications in bio-magnetic measurement due to their high sensitivity and miniaturized size. In this paper, we propose a SERF-based magnetometer using 1 × 2 polarization-maintaining fiber (PMF) with single-beam parameter optimization. The impacts of temperature, pumping laser power, and modulation amplitude on the magnetometer’s response signal at the SERF regime are examined. Moreover, through the simulation of zero-field resonance, the compensation accuracy is optimized. To further improve the compensation stability and accuracy, a novel finite state machine (FSM)-assisted iterative optimization magnetic field compensation algorithm is proposed. A pT-level compensation resolution with an error below 1.6% is achieved, which lays the foundation for the subsequent application of biomagnetic measurement arrays.

## 1. Introduction

Due to the widespread demand for highly accurate magnetic field measurements in various sectors, the sensitivity of magnetic field sensors has been continuously improved. Among these magnetic sensors are magnetometers with nitrogen-vacancy centers in diamond, fluxgate magnetometers, super quantum interfering effect (SQUID) magnetometers, and optically pumped atomic magnetometers [1]. Of these, the latter two types are capable of providing fT-level magnetic field sensitivity [2,3,4,5]. Despite its widespread application in bio-magnetic signal detection [6], SQUID magnetometers rely on chilling the sensor element with liquid helium or liquid nitrogen [7,8], which results in a huge volume and high costs. With the advancement of tunable semiconductor laser technology, lock-in amplifier technology, etc., the sensitivity of atomic magnetometers has been significantly promoted in weak magnetic sensing within the spin-exchange relaxation-free (SERF) regime [9,10,11]. In addition, it is also much easier to miniaturize the sensor to construct a sensor array, which is very crucial in biomedical applications, such as magnetocardiography [12], magnetoencephalography [13], and biomarker detection of magnetic markers [14,15].

For weak magnetic field measurement, it is a prerequisite to shield the environmental magnetic signal [16]. However, the remanent magnetic field inside the cylinder still approaches several tens of nanoteslas after shielding. Moreover, the electric heating system used in the SERF-based magnetometer also generates extra magnetic noise. Consequently, the establishment of a three-axis remanent magnetic field compensation system is required. Seltzer et al. realized that the SERF-based magnetometer operates a near-zero field using the cross-field modulation technique [17]. Li et al. measured the multicomponent field of magnetometers in a shielded environment using the z-axis parameter modulation approach [18]. Fang et al. proposed a modulation compensation method considering the pumping rate of the probe beam [19]. Wang et al. introduced a method using a modulated magnetic field to compensate for the combination of the DC remanent magnetic field and the gradient remanent magnetic field [20]. These methods not only have complex modulation and demodulation procedures but also introduce magnetic field cross-talk among three axes. Zhao et al. proposed a non-modulated three-axis magnetic compensation technique based on zero-field resonance to avoid the cross-talk effect [21]. Due to fundamental differences in the steady-state signal characteristics between double-beam and single-beam SERF-based magnetometers, the above magnetic compensation methods are applicable only to double-beam SERF-based magnetometers, while the single-beam scheme is widely used to realize miniaturization. To address compensation challenges in single-beam systems, Zhang et al. proposed a modulation and demodulation compensation method based on the steady-state response of single-beam SERF-based magnetometers [22]. However, this method does not support automated operation, and the accuracy is limited by low compensation efficiency. To improve performance, Dong et al. developed an autonomous compensation method for SERF-based magnetometers [23], while Long et al. introduced the trisection algorithm to enhance compensation efficiency [24]. Nevertheless, these methods failed to consider the triaxial non-orthogonal crosstalk and showed limited stability.

In this paper, we propose an FSM-assisted Iterative Optimization Magnetic Compensation Algorithm (FSM-IOMCA). By using the idea of a finite state machine (FSM), the complex magnetic field compensation process is decomposed into well-defined states and state transitions, which improves the stability, efficiency, and reliability of weak biomagnetic measurement. Additionally, the compensation speed, crosstalk effects, and compensation accuracy are improved by using an iterative optimization algorithm. Experiments show that the sensitivity of the magnetic compensation system in this paper is highly improved, even 38% higher than that of the Improved Hill-Climbing Algorithm (IHCA).

## 2. Principle

**Figure 1:**
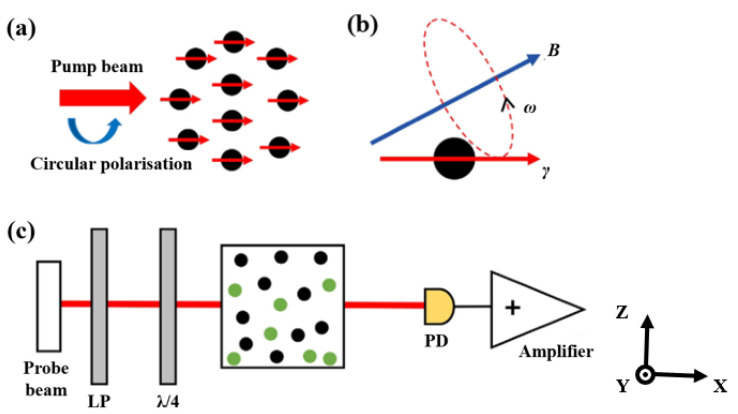
Principle diagram of SERF-based atomic magnetometer. (**a**) Atomic spin polarization; (**b**) Larmor precession; (**c**) Single-beam structure (LP: linear polarizer; PD: photoelectric detector).

As illustrated in Figure 1a, when a circularly polarized pump laser is resonant with the D1 transition of alkali metal atoms, the angular momentum of photons is transferred to the atomic spin system through resonant absorption. This interaction polarizes the initially random atomic spins, aligning them along the direction of the pump laser. In the presence of an external magnetic field ***B***, the polarized atomic spins undergo Larmor precession at a frequency $\omega =\gamma_{e}B$, where $\gamma_{e}$ denotes the alkali atom gyromagnetic ratio, as shown in Figure 1b. Since the spin angular momentum of a single atom is minimal, atomic ensembles are commonly employed to enhance the signal [25]. The Larmor precession induced by the magnetic field alters the collective polarization direction of the ensemble, which can be monitored by measuring changes in the transmitted laser’s absorption (Figure 1c).

In the 1970s, Happer et al. discovered that when the spin-exchange rate is much larger than the Larmor advance frequency, the spin-exchange broadening disappears and the spin-exchange relaxation is suppressed [26,27]. When the atomic ensemble operates under the SERF regime, the pumping and relaxation processes of atomic spins exist simultaneously. At this point, the Bloch equation can aptly describe the evolution of alkali metal atomic spins [25]. When defining the direction of the pump laser as the x-axis, the Bloch equation describing the atomic spin can be expressed as:

(1) $$\frac{dP}{dt}=\frac{1}{Q(P)}[\gamma_{e}B\times P+R_{op}(s\hat{x}-P)-R_{rel}P]$$

where ***P*** = [*P_x_*, *P_y_*, *P_z_*]^T^ represents the atomic polarization vector and its projections in three directions. *R_op_* is the pumping rate and *R_rel_* is the relaxation rate. *Q*(*P*) denotes the nuclear spin deceleration factor, and ***s*** denotes the laser pumping vector, which is aligned with the direction of laser pumping, and for the circularly polarized laser, |***s***| = 1. The deceleration factor *Q*(*P*) arises from the coupling between nuclear and electronic hyperfine levels, which slows down the precession of electrons. Table 1 presents the expressions for *Q*(*P*) corresponding to various nuclear spin quantum numbers. The parameters *q_lp_* and *q_hp_* represent the lower and upper bounds of *Q*(*P*) under low polarization (***P*** = 0) and high polarization (***P*** = 1) conditions, respectively.

When the external magnetic field varies slowly, such that dP/dt≈0, the system can be considered to be in a steady state. Under this condition, the steady-state solutions for atomic polarization components along three directions can be derived as:

(2) $$\left\{\begin{array}{c} P_{x}=\frac{R_{op}}{R_{op}+R_{rel}}\frac{{B_{x}}^{2}+{\left[\left(R_{op}+R_{rel}\right)/\gamma_{e}\right]}^{2}}{{B_{z}}^{2}+{B_{y}}^{2}+{B_{x}}^{2}+{\left[\left(R_{op}+R_{rel}\right)/\gamma_{e}\right]}^{2}}\\ P_{y}=\frac{R_{op}}{R_{op}+R_{rel}}\frac{B_{x}B_{y}-\left[\left(R_{op}+R_{rel}\right)/\gamma_{e}\right]B_{z}}{{B_{z}}^{2}+{B_{y}}^{2}+{B_{x}}^{2}+{\left[\left(R_{op}+R_{rel}\right)/\gamma_{e}\right]}^{2}}\\ P_{z}=\frac{R_{op}}{R_{op}+R_{rel}}\frac{B_{x}B_{z}+\left[\left(R_{op}+R_{rel}\right)/\gamma_{e}\right]B_{y}}{{B_{z}}^{2}+{B_{y}}^{2}+{B_{x}}^{2}+{\left[\left(R_{op}+R_{rel}\right)/\gamma_{e}\right]}^{2}} \end{array}\right.$$

For a single-beam SERF-based magnetometer, the photoelectric signal *P_PD_* is proportional to the atomic polarization component along the pump direction, which contains information about the three-axis magnetic field [28,29]. In this paper, the pump beam is along the x-direction; consequently, the *P_PD_* varies consistently with the polarization component *P_x_*. The remanent magnetic field can be accurately measured based on the steady-state solution of *P_x_*, which is known as zero-field resonance (ZFR) [24].

## 3. Simulation

**Figure 2:**
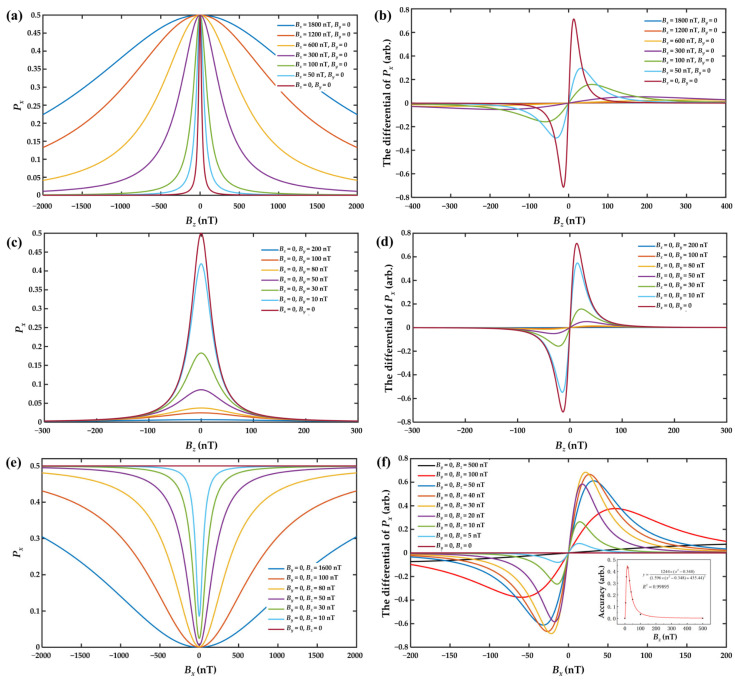
(**a**) *B_z_*-*P_x_* relationship simulation under different *B_x_*; (**b**) dispersion response of *B_z_* under different *B_x_*; (**c**) *B_z_*-*P_x_* relationship simulation under different *B_y_*; (**d**) dispersion response of *B_z_* under different *B_y_*; (**e**) *B_x_*-*P_x_* relationship simulation under different *B_z_*; (**f**) dispersion response of *B_x_* under different *B_z_* (the lower-right graph displayed the maximum fitting accuracy of *B_x_* response slope).

To provide an intuitive understanding of the ZFR phenomenon, the relationship between the triaxial magnetic field and atomic polarization component *P_x_* was investigated through a simulation based on Equation (2). The simulation parameters were set as *R_op_* = *R_rel_* = 500 s^−1^ and $\gamma_{e}$≈ 2π × 6.996 GHz/T. As illustrated in Figure 2, the dependence of *P_x_* on the triaxial magnetic field exhibited absorption-like line shapes. By differentiating these curves, the dispersion-like response signal was obtained, and subsequently normalized for analysis. In this paper, the x-axis was the pumping direction and the external magnetic field ***B*** could be divided into the longitudinal magnetic field *B_x_* and the transverse magnetic fields *B_y_* and *B_z_*.

As shown in Figure 2a,c, the atomic polarization component *P_x_* reached a maximum when *B_z_* = 0. This indicated that to compensate for the remanent magnetic field along the z-axis, it was necessary to adjust the current in the z-direction magnetic field coil until the photoelectric signal *P_PD_* was maximized. From Figure 2a, it can be observed that variations in the longitudinal magnetic field *B_x_* only influenced the resonance linewidth, without affecting the peak amplitude of the *P_x_* response. This phenomenon could be attributed to the fact that the existence of a longitudinal magnetic field broke the energy-level degeneracy under the SERF regime, increasing the splitting between atomic energy levels. This splitting introduced an additional relaxation mechanism, thereby enhancing the overall spin relaxation rate and broadening the resonance linewidth. As depicted in Figure 2b, smaller values of *B_x_* correspond to steeper slopes in the dispersion response, resulting in higher compensation accuracy along the z-axis. Conversely, Figure 2c demonstrates that changes in the transverse magnetic field *B_y_* only affected the amplitude of the *P_x_* response, with no significant impact on the linewidth. This was because, under the SERF regime, atomic spins were predominantly polarized along the pumping direction (x-axis). A transverse magnetic field altered the precession direction of spins, thereby reducing the component of atomic polarization along the pumping direction. Similarly, Figure 2d shows that decreasing *B_y_* leads to an increase in the slope of dispersion response, enhancing the accuracy of z-axis magnetic field compensation. Therefore, to achieve optimal compensation accuracy along the z-axis, it was necessary to zero both *B_x_* and *B_y_*. The y- and z-axes, being orthogonal to the pump axis, exhibited similar behaviors in this context.

As shown in Figure 2e, the atomic polarization component *P_x_* reached a minimum when *B_x_* = 0. This indicated that to compensate for the remanent magnetic field along the x-axis, it was necessary to adjust the current in the x-direction magnetic field coil until the photoelectric signal *P_PD_* was minimized. However, a phenomenon was observed during the x-axis compensation process: when the magnetic fields along the y- and z-axes were simultaneously compensated to zero (*B_y_* = *B_z_* = 0), the absorption-like resonance signal along the pumping direction (x-axis) disappeared, thereby preventing effective compensation of the x-axis remanent magnetic field. This phenomenon occurred because, in the absence of transverse magnetic fields, the polarization direction of atomic spins aligned with that of the pump laser, resulting in maximum atomic polarization. Consequently, the interaction between the pump laser and the atomic spins became saturated, and the absorption of the pump laser by atoms ceased to depend on changes in the magnetic field. To address this problem, a small offset magnetic field had to be applied along the y- and z-axes. The compensation accuracy in the x-direction was quantified by extracting the slope of an approximately linear region near-zero field in the x-direction dispersion response, as shown in Figure 2f. This slope reflected the sensitivity of the overall system to variations in *B_x_* and thus served as a measure of compensation accuracy. The fitted curve in the lower-right graph of Figure 2f demonstrated that the x-axis compensation accuracy initially increased, reached a maximum, and then decreased with the applied transverse magnetic field. The optimal compensation was achieved when *B_y_* = *B_z_* = 17 nT.

## 4. System Design

**Figure 3:**
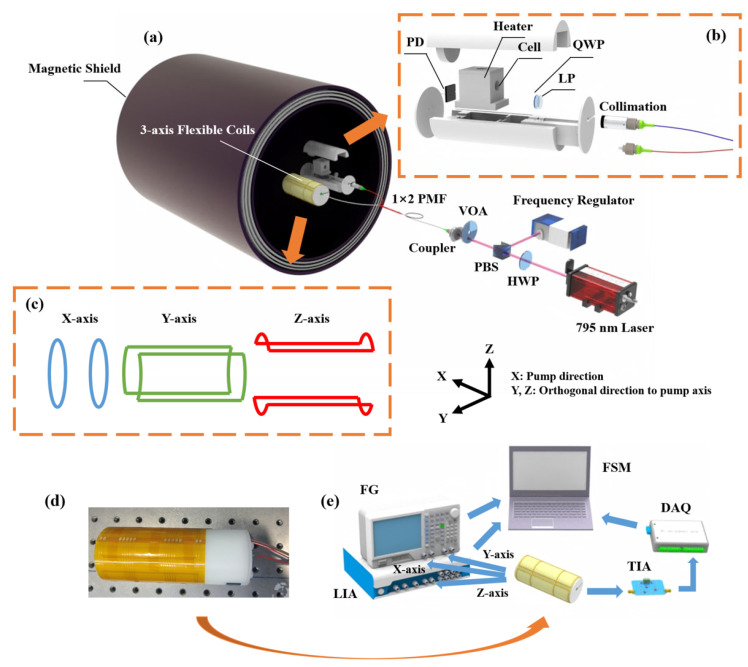
System design of the dual-channel single laser beam SERF-based magnetometer. (**a**) Overall structure for dual-channel SERF-based magnetometer. The ^87^Rb vapor cell was situated in the middle of five layers of magnetic shield cylinder. The pump laser, oriented along the x-direction, was frequency-detuned from the D1 transition of Rb. (**b**) The SERF-based magnetometer probe’s internal construction. Spatial laser was coupled into the dual-channel probe using a 1 × 2 PMF. (**c**) The structure of three-axis coil system. (**d**) The physical structure of SERF-based magnetometer probe. (**e**) Three-axis coil control and feedback system. Each module was controlled by FSM to achieve automation. (HWP: half-wave plate; PBS: polarization beam splitter; VOA: variable optical attenuator; PMF: polarization-maintaining fiber; LP: linear polarizer; QWP: quarter-wave plate; PD: photodiode; FG: function generator; LIA: lock-in amplifier; TIA: transimpedance amplifier; DAQ: data acquisition card; FSM: finite state machine).

As illustrated in Figure 3, an experimental setup was constructed to validate the effectiveness of the proposed magnetic compensation method. The overall structure of the dual-channel SERF-based magnetometer is illustrated in Figure 3a. A 795 nm external cavity diode laser (ECDL) served as both the pump and probe laser. The laser beam was divided into two beams, and its ratio was adjusted between laser frequency stabilization and the vapor cell using a half-wave plate (HWP) and polarization beam splitter (PBS). The laser frequency stabilization was used to stabilize the laser frequency at the atomic saturation absorption peak of the rubidium atom’s D1 line. A variable optical attenuator (VOA) was employed to regulate the incident laser power. A coupler and a 1 × 2 polarization-maintaining fiber (PMF) were used to couple the spatial laser with two magnetometer probes. Figure 3b displays the internal construction of the SERF-based magnetometer probe. When the PMF was exposed to temperature fluctuations or external stress, its birefringence axis underwent rotation, resulting in a drift in the output polarization state. Consequently, the laser beam emerging from the collimator might deviate from an ideal linear polarization. To ensure the desired polarization state for effective optical pumping, a linear polarizer (LP) was introduced after the collimator, and a quarter-wave plate (QWP) was used to generate a circularly polarized laser beam. ^87^Rb atoms and 300 Torr N_2_ buffer gas were filled in the 10 mm diameter vapor cell. A 20 kHz alternating current was employed to heat the vapor cell to the temperature of 120 °C to 140 °C. With the use of the PT100 resistance temperature detector (RTD) and the PID controller, a real-time temperature control system was created that achieved temperature stability with fluctuations maintained below 0.82 °C. A photodiode (PD) was used to convert optical signals into electrical signals. The magnetic compensation system consisted of a magnetic shielding cylinder for passive shielding from the massive geomagnetic field and three-axis coils for active compensation from remanent magnetic field interference. Among them, the magnetic shielding cylinder was constructed from four layers of mu-metal and one layer of Mn-Zn ferrite. The structure of the three-axis coil system is illustrated in Figure 3c, which includes a pair of circular Helmholtz coils aligned along the axial direction of the cylindrical probe, generating a uniform magnetic field along the sensor’s primary sensitive axis (x-axis). Additionally, two pairs of saddle-shaped coils were positioned orthogonally to produce magnetic fields in the transverse directions (y- and z-axes).

Figure 3d illustrates the physical structure of the SERF-based magnetometer probe. The three-axis coil system was fabricated on a flexible printed circuit (FPC) board, allowing it to conform precisely to the contours of the probe. This design enhanced system integration while also avoiding the geometric deviations introduced by manually wound coils, reducing crosstalk, and improving magnetic field uniformity. The magnetic compensation algorithm primarily relied on the system shown in Figure 3e, where a two-channel function generator (FG, RIGOL DG4000, China) drove the x- and y-axes coils and a lock-in amplifier (LIA, CIQTEK LIA001M, China) drove the z-axis coil. We also connected a high-precision 50 kΩ resistor in series with each coil; the coil constant calibration values for the x-uniform coil and y-uniform coil were 27.06 nT/mA and 20.63 nT/mA, respectively, while the coil constant calibration for z-uniform coil was 41.54 nT/mA. A transimpedance amplifier (TIA, China) was employed to convert the photogenerated currents into voltage signals suitable for subsequent signal processing. The DC bias during optical absorption method detection was eliminated by a PI controller [30], and high-frequency noise was reduced by a low-pass filter. A data acquisition card (DAQ) facilitated the real-time feedback of photodetector signals. FG, LIA, and DAQ were coordinated by a finite state machine (FSM) system to automate the magnetic field compensation procedure.

## 5. Magnetic Compensation Method

### 5.1. Finite State Machine Model of Three-Axis Autonomous Magnetic Field Compensation System

The finite state machine (FSM), derived from the theory of finite automata, comprises a finite set of well-defined states with transitions between them triggered by specific events [31,32]. At any given moment, the system resides in only one of these states. This architecture provides an efficient, stable, and reliable approach for managing complex, sequential operations in embedded and control systems.

**Figure 4:**
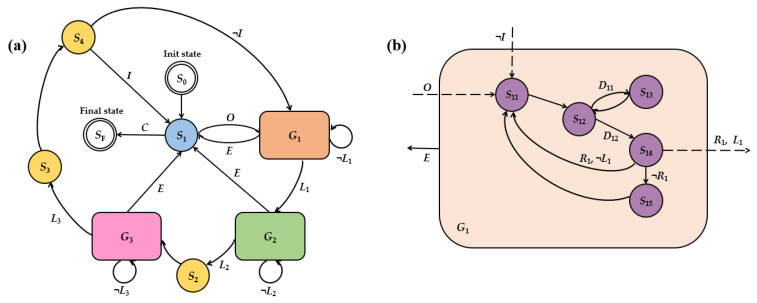
(**a**) The state diagram of three-axis autonomous magnetic compensation algorithm. (**b**) Single-axis magnetic compensation state diagram, taking the z-axis as an example (*G*_1_). The sub-states of *G*_2_ and *G*_3_ follow a structure similar to that of *G*_1_, where the only distinction lies in sub-state *S*_35_ of *G*_3_ state set, which implements an inverse decision logic compared to sub-states *S*_15_ (in *G*_1_) and *S*_25_ (in *G*_2_). Specifically, in *S*_35_, if the photodetector signal at *L_x_* exceeds that at *R_x_* (i.e., *PD*(*L_x_*) > *PD*(*R_x_*)), the system sets the driving current as *I_x_* = *R_x_*.

The FSM serves as a model to describe the dynamic behavior of a system through discrete state transitions, typically represented by a state diagram (SD). This graphical representation provides an intuitive overview of the system’s operation and transition logic. The state diagram of the three-axis autonomous magnetic compensation algorithm is shown in Figure 4 and comprises five primary states: Waiting, Z-axis Compensation, Y-axis Compensation, X-axis Compensation, and Sensitivity Enhancement. The Waiting state (blue) was responsible for establishing communication with FG, LIA, and DAQ while configuring initial parameters and controlling start/stop functionality. The Z-axis Compensation states (orange) identified the maximum value of the photoelectric signal *P_PD_* and adjusted the z-axis magnetic field coil to compensate for the remanent magnetic field. Similarly, the Y-axis Compensation states (green) identified the maximum value of the photoelectric signal *P_PD_* and adjusted the y-axis magnetic field coil to compensate for the remanent magnetic field. In contrast, the X-axis Compensation states (pink) identified the minimum value of the photoelectric signal *P_PD_* and adjusted the x-axis magnetic field coil to compensate for the remanent magnetic field. The Sensitivity Enhancement states (yellow) further refined the accuracy of three-axis compensation. Each axis compensation state comprised five sub-states (purple), with Figure 4b illustrating the detailed structure of Z-axis Compensation states.

The FSM can be represented as a functional group consisting of five elements [33], as follows:

(3) $$M=\left(Q,\Sigma ,\delta ,q_{0},F\right)$$

where *Q* denotes a finite set of states and Σ denotes a finite set of input events. The state transition function *δ*: *Q* × Σ → *Q* defines how the FSM transitions between states based on input events. Specifically, for a given state and input event, the FSM moves to a new state determined by *δ*. $q_{0}\in Q$ is the initial state, from which the FSM starts to process input events. F⊆Q is the set of final states, which are states that, once reached, prevent further input events from being processed by the FSM. In this paper, FSM can be represented as:

$$Q=\left\{S_{0},\;S_{1},\;G_{1},\;G_{2},\;G_{3},\;S_{2},\;S_{3},\;S_{4}\right\}$$

$$\begin{array}{l} \Sigma =\left\{O,\;C,\;E,\;L_{1},\;\neg L_{1},\;L_{2},\;\neg L_{2},\;L_{3},\;\neg L_{3},\;I,\;\neg I,\right.\;\\ \left.\left\{D_{11},\;D_{12},\;R_{1},\;\neg R_{1}\right\},\;\left\{D_{21},\;D_{22},\;R_{2},\;\neg R_{2}\right\},\;\left\{D_{31},\;D_{32},\;R_{3},\;\neg R_{3}\right\}\right\} \end{array}$$

$$\begin{array}{ll} \delta = & \left\{\left(S_{1},O,S_{11}\right),\;\left(S_{1},C,S_{F}\right),\;\left(G_{1},E,S_{1}\right),\;\left(G_{2},E,S_{1}\right),\;\left(G_{3},E,S_{1}\right),\;\left(S_{4},I,S_{1}\right),\;\left(S_{4},\neg I,S_{11}\right),\right.\\ & \left(S_{12},D_{11},S_{13}\right),\;\left(S_{12},D_{12},S_{14}\right),\;\left(S_{14},\left\{R_{1},L_{1}\right\},S_{21}\right),\;\left(S_{14},\left\{R_{1},\neg L_{1}\right\},S_{11}\right),\;\left(S_{14},\neg R_{1},S_{15}\right),\\ & \left(S_{22},D_{21},S_{23}\right),\;\left(S_{22},D_{22},S_{24}\right),\;\left(S_{24},\left\{R_{2},L_{2}\right\},S_{2}\right),\;\left(S_{24},\left\{R_{2},\neg L_{2}\right\},S_{21}\right),\;\left(S_{24},\neg R_{2},S_{25}\right),\\ & \left.\left(S_{32},D_{31},S_{33}\right),\;\left(S_{32},D_{32},S_{34}\right),\;\left(S_{34},\left\{R_{3},L_{3}\right\},S_{3}\right),\;\left(S_{34},\left\{R_{3},\neg L_{3}\right\},S_{31}\right),\;\left(S_{34},\neg R_{3},S_{35}\right)\right\} \end{array}$$

$$q_{0}=S_{0}$$

(4) $$F=\left\{S_{F}\right\}$$

where $G_{1}=\left\{S_{11},S_{12},S_{13},S_{14},S_{15}\right\}$, $G_{2}=\left\{S_{21},S_{22},S_{23},S_{24},S_{25}\right\}$, $G_{3}=\left\{S_{31},S_{32},S_{33},S_{34},S_{35}\right\}$.

The input events that trigger state transitions may include *Y* and ¬*Y*, where the symbol ¬ represents logical negation. Accordingly, ¬*Y* denotes the logical complement of *Y*, or more precisely, the condition “not *Y*.” These input events govern transitions between states in the finite state machine. The states and transitions illustrated in Figure 4 are described in detail below:

- *S*_0_ (Init state): This state initializes all relevant system parameters before entering the compensation routine.
- *S*_1_ (Waiting state): In this state, the system awaits events input:—either “open” (*O*), which initiates the magnetic compensation process, transitioning the system to the compensation states, or “close” (*C*), which stops the magnetic compensation process, transitioning the system to the final state *S_F_*.
- *G*_1_ (Z-axis Compensation states): This set includes sub-states dedicated to Z-axis compensation. Two photodetector signals, denoted as *L_z_* and *R_z_*, are sampled and analyzed. If the absolute difference between two samples is within the predefined minimum allowable error threshold *ε_z_* (*L*_1_), the system transitions to *G*_2_, the Y-axis compensation states; if the absolute difference exceeds the minimum error threshold *ε_z_* (¬*L*_1_), the step size *l_z_* and error threshold *E_z_* are reduced, and *G*_1_ is re-executed; if an execution error (*E*) occurs any sub-state, the system transitions back to *S*_1_, awaiting further events input.
- *G*_2_ (Y-axis Compensation states): The process mirrors *G*_1_, with two signals *L_y_* and *R_y_* sampled and analyzed. If *L*_2_ is satisfied (within minimum error threshold *ε_y_*), the system transitions to *S*_2_; if not (¬*L*_2_), the step size *l_y_* and error threshold *E_y_* are reduced, and *G*_2_ is re-executed. In the case of an error (*E*), the system returns to *S*_1_.
- *G*_3_ (X-axis Compensation states): Follows a process analogous to *G*_1_ and *G*_2_, using samples *L_x_* and *R_x_*. If within a minimum error threshold *ε_x_* (*L*_3_), transition to *S*_3_. Otherwise, we reduce *l_x_* and *E_x_*, and re-execute *G*_3_. Errors (*E*) lead to a transition to *S*_1_.
- *S*_2_ (Addition state): Applies a magnetic field increment of 17 nT in both y- and z-directions.
- *S*_3_ (Reduction state): Reverts the 17 nT magnetic field increment applied in *S*_2_, restoring y- and z-directions to their original values.
- *S*_4_ (Cycle state): Determines whether to perform additional compensation cycles. If the cycle index reaches 3 (*I*), the compensation process is deemed complete and the system returns to *S*_1_; if not (¬*I*), the initial step size *l* and error threshold *E* are reduced, and the system re-enters *G*_1_ to begin another compensation cycle.
- *S_F_* (Final state): Terminates the compensation process.

Sub-States of *G*_1_:

- *S*_11_: Decreases the z-direction coil current *I_z_* by a step *l_z_*, setting *L_z_* = *I_z_* − *l_z_*.
- *S*_12_: Samples photodetector signals. The “first sample” (*D*_11_), taken at *L_z_*, is recorded as *PD*(*L_z_*). The system then proceeds to *S*_13_. The “second sample” (*D*_12_), taken at *R_z_*, is recorded as *PD*(*R_z_*), then proceeds to *S*_14_.
- *S*_13_: Increases *I_z_* by a step *l_z_*, setting *R_z_* = *I_z_* + *l_z_*.
- *S*_14_: Computes the error as | *L_z_* − *R_z_* |/2. If both the error threshold *E_z_* (*R*_1_) and minimum error threshold *ε_z_* (*L*_1_) are met, transition to *G*_2_; if only *R*_1_ is satisfied but not *L*_1_, reduce *l_z_* and *E_z_*, and return to *S*_11_; if *R*_1_ is not satisfied, transition to *S*_15_.
- *S*_15_: Compares the sampled values. If *PD*(*L_z_*) > *PD*(*R_z_*), set *I_z_* = *L_z_*; Otherwise, set *I_z_* = *R_z_*.

### 5.2. Iterative Optimization Method of Three-Axis Autonomous Magnetic Field Compensation System

**Figure 5:**
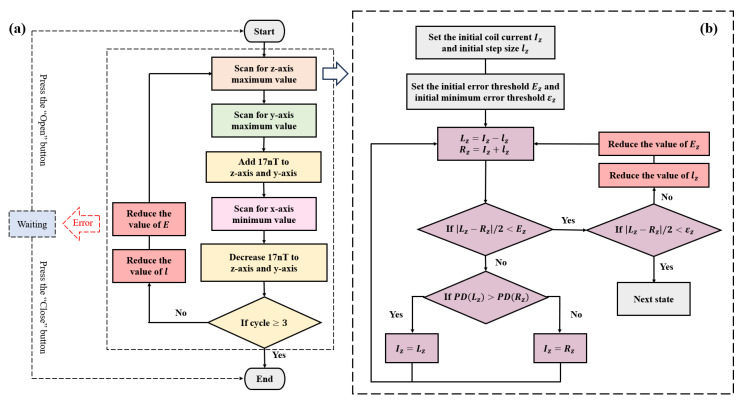
(**a**) Flowchart of three-axis autonomous magnetic field compensation system. (**b**) Uniaxial compensation method, using the z-axis as an example. The y-axis followed the same logic, while x-axis operated in the opposite manner. Specifically, when *PD*(*L_x_*) > *PD*(*R_x_*), the compensation current *I_x_* = *R_x_*.

To provide a more intuitive understanding of compensation method implementation, a flowchart is presented in Figure 5. The flowchart maintains a consistent color scheme with the state diagram to facilitate correspondence between the two.

Based on the principles (Section 2) and simulation results (Section 3) of three-axis autonomous magnetic field compensation, the remanent magnetic field was zero when the photodetector signal *P_PD_* reached its minimum along the pump direction (x-axis) and its maximum along the orthogonal directions (y- and z-axes). As illustrated in Figure 5b, the z-axis was used as an example to demonstrate the uniaxial compensation process. Initial values were set to ensure system stability in each operation. The maximum value of the *P_PD_* signal was determined using an improved hill-climbing algorithm through iterative optimization. During the compensation process, the step size *l_z_* was progressively reduced until the error fell below the predefined minimum threshold *ε_z_*, thereby enhancing uniaxial compensation accuracy.

To reduce environmental interference, the system averaged ten sets of *P_PD_* signals and applied wavelet-based denoising to filter out high-frequency noise. Due to substantial noise generated by the heating system during the heating phase—which could cause the *P_PD_* signal to drop to zero—only signals measured during the heat-preservation phase were considered valid. Specifically, data points where *PD*(*L_z_*) and *PD*(*R_z_*) were below 0.5 V were excluded from the analysis.

As shown in Figure 5a, to improve the compensation accuracy along the x-axis, a 17 nT auxiliary magnetic field was initially applied along the y- and z-axes, as indicated by the simulation results in Figure 2f. After completing the x-axis compensation, the auxiliary magnetic field was removed. To improve the compensation accuracy along the y- and z-axes, multiple iterative cycles were performed to minimize the remanent magnetic field. In the initial iteration, a larger step size was adopted to rapidly converge toward the optimal compensation region, thereby improving efficiency. A common challenge in magnetic field compensation is the non-orthogonal arrangement of three-axis coils, which introduced inter-axis crosstalk [21]. Furthermore, in the case of the dual-channel magnetometer used in this paper, a large initial step size might exacerbate magnetic field crosstalk between two magnetometer probes. To mitigate these effects, the system progressively reduced the step size *l* during each compensation cycle. This approach not only shortened the overall compensation time but also effectively reduced inter-axis and inter-probe crosstalk and simultaneously enhanced overall compensation accuracy.

The FSM-assisted Iterative Optimization Magnetic Compensation Algorithm (FSM-IOMCA) proposed in this paper offered several distinct advantages over previously reported schemes. The Traditional Hill-Climbing Algorithm (THCA) performed one-pass, three-axis magnetic field compensation using fixed initial parameters (step size *l* and error threshold *E*), without parameter adjustment during the compensation process. In contrast, the Improved Hill-Climbing Algorithm (IHCA) incrementally reduced both the step size *l* and error threshold *E* during uniaxial compensation to approach the minimum error threshold *ε*. Nonetheless, IHCA performed only a single-cycle sequential three-axis compensation, which limited its precision. The Iterative Optimization Magnetic Compensation Algorithm (IOMCA) introduced in this work adopted an iterative optimization approach to enhance the compensation accuracy. It enhanced the compensation accuracy of each axis through multiple compensation cycles, gradually decreasing both the step size *l* and error threshold *E* within each single-axis optimization, ultimately achieving the minimum error threshold *ε*. Across the complete compensation process, three iterative cycles were executed, with the initial step size *l* progressively reduced in each cycle. This iterative approach—combining a coarse search using large step sizes with fine compensation using smaller steps—not only improved overall compensation accuracy but also effectively reduced inter-axis and inter-probe crosstalk. Furthermore, the proposed IOMCA method incorporated optimizations derived from the SERF-based magnetometer principle, which significantly improved its three-axis magnetic compensation accuracy.

The Manual Magnetic Compensation Algorithm (MMCA) required continuous manual adjustment of coil currents along each axis and subjective monitoring of oscilloscope outputs. This process was inherently time-consuming, prone to human error, and resulted in low compensation accuracy. In contrast, FSM-IOMCA fully automated the compensation process. The finite state machine design ensured that state transitions follow a deterministic rule δ: Q × Σ → Q, where each transition was explicitly defined to minimize delays and achieve microsecond-level response times. The decomposition of the algorithm into discrete FSM states eliminated redundant computations, and the inclusion of a global fault-tolerant mechanism ensured graceful degradation in case of anomalies, thereby enhancing system stability, efficiency, and reliability. As a result, FSM-IOMCA was suitable for high-accuracy and autonomous magnetic field compensation in SERF-based magnetometer systems.

## 6. Experimental Results

### 6.1. Parameters Optimization of SERF-Based Magnetometer

**Figure 6:**
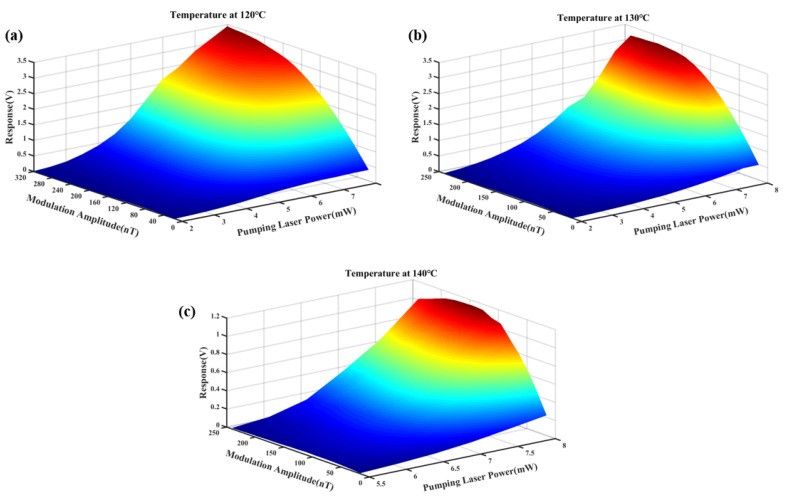
The responses of SERF-based magnetometers at different pumping laser powers and modulation magnetic field amplitudes at (**a**) 120 °C, (**b**) 130 °C, and (**c**) 140 °C.

To achieve optimal sensitivity in the SERF-based magnetometer following the magnetic compensation process described above, key parameters such as temperature, pumping laser power, and modulation magnetic field amplitude had to be optimized [21]. In this paper, we studied the combined effects of the pumping laser power and modulation amplitude on the response of the SERF-based magnetometer at three operating temperatures: 120 °C, 130 °C, and 140 °C, as illustrated in Figure 6.

In the experimental setup, a sinusoidally modulated magnetic field at a frequency of 1 kHz was applied along the z-axis, and the magnetometer response was extracted via the LIA. Due to the experimental limitation imposed by the 1 × 2 PMF, the maximum available pumping laser power was restricted to 8 mW. At an operating temperature of 140 °C, the elevated atomic vapor density within the cell resulted in the complete absorption of the transmitted laser at lower power levels. Consequently, measurements at this temperature commenced at a minimum pumping laser power of 5.5 mW to ensure signal detectability.

The experimental results revealed that the optimal pumping laser power was consistently 8 mW across all three temperatures, and this consistency might be attributed to the experimental platform’s output power limitation. Moreover, the optimal modulation amplitude decreased with increasing temperature: at 120 °C, the optimal modulation amplitude was 320 nT; at 130 °C, it was 192 nT; and at 140 °C, it decreased further to 144 nT. This inverse relationship between temperature and optimal modulation amplitude could be explained by the behavior of the optical pumping rate (*R_OP_*) and the relaxation rate (*R_rel_*). As the temperature decreased, *R_OP_* rose sharply and might exceed *R_rel_*, disrupting the balance required for maximum polarization. The optimal SERF-based magnetometer response was achieved when *R_OP_* ≈ *R_rel_*. Increasing the modulation amplitude raised *R_rel_* [34], reducing the difference between *R_OP_* and *R_rel_*, which in turn enhanced the magnetometer response.

As shown in Figure 6, the maximum magnetometer response was achieved when the temperature was 120 °C, the pumping laser power was 8 mW, and the modulation magnetic field amplitude was 320 nT. Therefore, these parameters were selected for subsequent experiments to optimize the overall performance and sensitivity of the SERF-based magnetometer.

**Figure 7:**
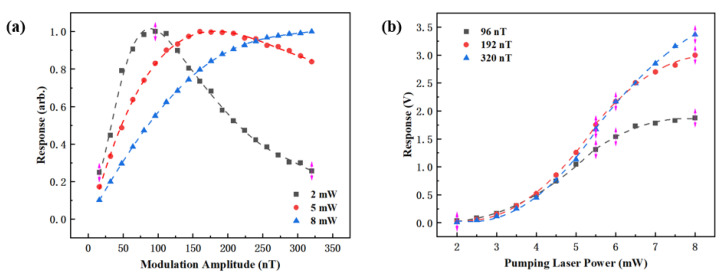
(**a**) Normalized response for various modulation amplitudes at three pumping laser powers; (**b**) dispersion response for various pumping laser powers at three modulation amplitudes.

To further investigate the relationship between the pumping laser power and modulation magnetic field amplitude, the magnetometer response data obtained at 120 °C were analyzed in greater detail. Specifically, the effect of the modulation magnetic field amplitude on the magnetometer response was extracted at three different incident pumping laser powers (2 mW, 5 mW, and 8 mW), as shown in Figure 7a. For consistency and ease of comparison, the response amplitudes were normalized.

The results indicated that the optimal modulation magnetic field amplitude increased with increasing incident pumping laser power. The corresponding optimal modulation amplitudes at 2 mW, 5 mW, and 8 mW were 96 nT, 160 nT, and 320 nT, respectively. Furthermore, the response curve near the optimal modulation amplitude became flatter at higher pumping laser powers. This indicated that the magnetometer’s response became less sensitive to small variations in the modulation amplitude when operating at a higher pumping laser power. This phenomenon was attributed to the corresponding increase in *R_OP_* with higher pumping laser power. As *R_OP_* increased, the relative contribution of the relaxation rate induced by the modulated magnetic field to *R_rel_* decreased. This reduction diminished the system’s sensitivity to modulation amplitude variations.

In addition, this study compared the magnetometer response at three different modulation amplitudes under varying pumping laser powers, as shown in Figure 7b. The experimental results showed that the response signal increased rapidly with pumping laser power at lower levels but gradually saturated as pumping laser power approached 8 mW. This suggested that absorption by the vapor cell reached saturation, limiting further increases in the response signal amplitude. At lower pumping laser power levels, a modulation amplitude of 96 nT yielded a higher response, while at a higher pumping laser power (e.g., 8 mW), a larger modulation amplitude of 320 nT produced a stronger signal, which further corroborated the findings from Figure 7a.

### 6.2. Performance Analysis of Magnetic Compensation Methods

**Figure 8:**
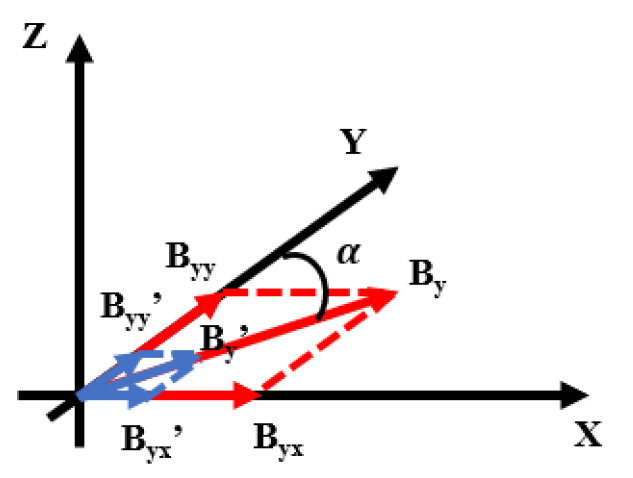
A non-orthogonal schematic diagram of the magnetic field coil between x-axis and y-axis. If there was a deviation of α between the actual position and the ideal position of y-axis coil, B_yy_ was the magnetic field projected B_y_ in the y-direction, and B_yx_ was the magnetic field projected by B_y_ in the x-direction; B_yy_′ was the magnetic field projected B_y_′ in the y-direction, and B_yx_′ was the magnetic field projected by B_y_′ in the x-direction.

As illustrated in Figure 8, the crosstalk phenomenon was analyzed using the y-axis as an example. When the step size was large, a significant magnetic field B_y_ was generated by the y-axis coil. If the coil’s actual position deviated from its ideal orientation by an angle α, the magnetic field B_y_ was decomposed into two components: B_yy_, aligned with the y-axis, and B_yx_, inadvertently projected along the x-axis. This projection of the y-axis magnetic field onto the x-axis resulted in magnetic crosstalk. Conversely, as the step size decreased, the generated magnetic field B_y_′ became smaller, and its corresponding projections B_yy_′ and B_yx_′ along the y- and x-directions were also reduced, thereby mitigating the crosstalk effect.

**Figure 9:**
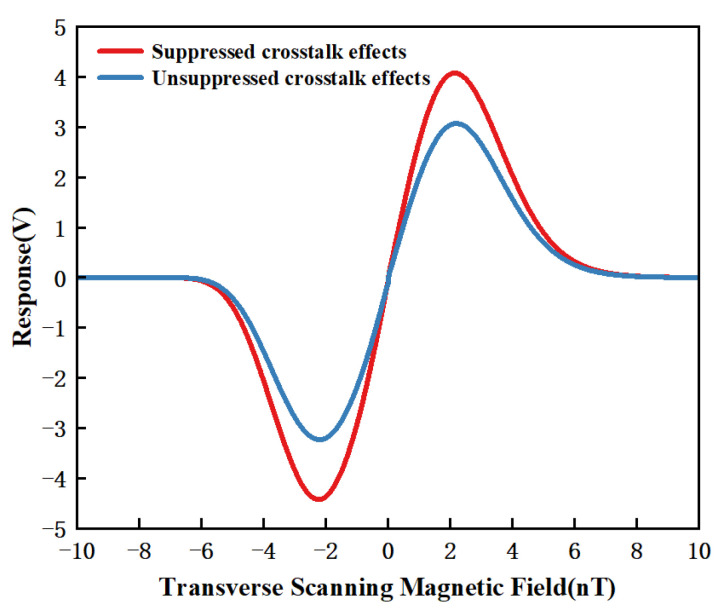
Zero-field dispersion curves under suppressed crosstalk effects and unsuppressed crosstalk effects.

The presence of remanent magnetic fields reduced the slope of the dispersion curve’s linear region, thereby degrading the sensitivity of the SERF-based magnetometer [35]. To evaluate the effectiveness of the proposed FSM-IOMCA method in suppressing crosstalk, we conducted a comparative experiment under a 1 Hz triangular wave signal, where the “suppressed crosstalk effects” utilized the FSM-IOMCA method proposed in this paper, while the “unsuppressed crosstalk effects” employed a fixed initial step size *l* and error threshold *E* over three cycles. The experimental results are shown in Figure 9, indicating that the proposed FSM-IOMCA method significantly suppressed crosstalk, effectively reducing the introduction of unnecessary magnetic fields on non-target axes during the compensation process and thereby improving the overall sensitivity of the magnetometer.

**Figure 10:**
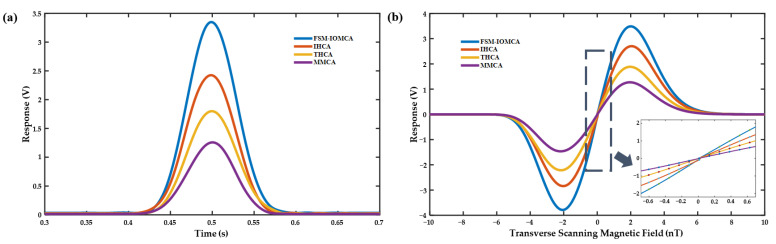
(**a**) Lorentz absorption curves for four compensation methods; (**b**) zero-field dispersion curves for four compensation methods (the lower-right graph displayed the linear response from −0.7 to 0.7 nT for easier comparison).

To evaluate the performance of four magnetic field compensation methods—FSM-IOMCA, IHCA, THCA, and MMCA—we evaluated them under consistent experimental conditions, using the previously optimized temperature, pumping laser power, and modulation magnetic field amplitude. A 1 Hz triangular wave signal was applied to scan the magnetic field, and the results are presented in Figure 10a. All four curves exhibited Lorentzian absorption line shapes, confirming that the magnetometer operated in the SERF regime. Figure 10b presents the corresponding dispersion curves obtained through LIA demodulation of the magnetometer output signals. In the near-zero magnetic field range, the dispersion curves were approximately linear response. Based on the slope of the dispersion curves, the magneto-voltage conversion ratios for the MMCA, THCA, IHCA, and FSM-IOMCA methods were calculated to be 1.02 V/nT, 1.49 V/nT, 2.12 V/nT, and 2.77 V/nT, respectively. These results clearly demonstrated that the FSM-IOMCA method achieved the highest signal response and compensation accuracy among the four methods, demonstrating its superior performance in enhancing the sensitivity of the SERF-based magnetometer.

Sensitivity is a critical performance metric for evaluating the effectiveness of three-axis remanent magnetic field compensation. A 2.5 nT_rms_ calibration magnetic field at a frequency of 30 Hz was applied to the system, and the response of the SERF-based magnetometer was recorded. The data acquisition function of LIA was employed to continuously record the signal over a duration of 10 s. Subsequently, the recorded data were processed using a Fast Fourier Transform (FFT) to obtain the power spectral density (PSD) of the system.

**Figure 11:**
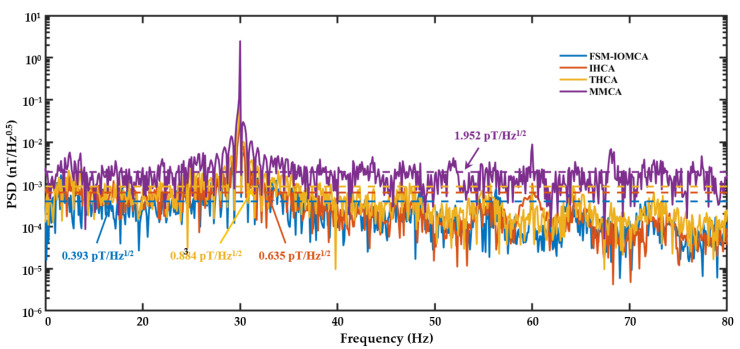
PSD of SERF-based magnetometer’s responses under four compensation methods: FSM-IOMCA (blue line), IHCA (orange line), THCA (yellow line), and MMCA (purple line). A calibration magnetic field signal of 2.5 nTrms in 30 Hz was utilized.

As illustrated in Figure 11, the measured sensitivities for the four compensation schemes—MMCA, THCA, IHCA, and FSM-IOMCA—were 1.952 pT/Hz^1/2^, 0.884 pT/Hz^1/2^, 0.635 pT/Hz^1/2^, and 0.393 pT/Hz^1/2^, respectively. These results demonstrated that the FSM-assisted iterative optimization magnetic compensation algorithm (FSM-IOMCA) achieved the highest sensitivity among the four methods. Specifically, the FSM-IOMCA method yielded an 80% improvement in sensitivity compared to MMCA, and sensitivity improvements of 38% and 55% over IHCA and THCA, respectively.

**Figure 12:**
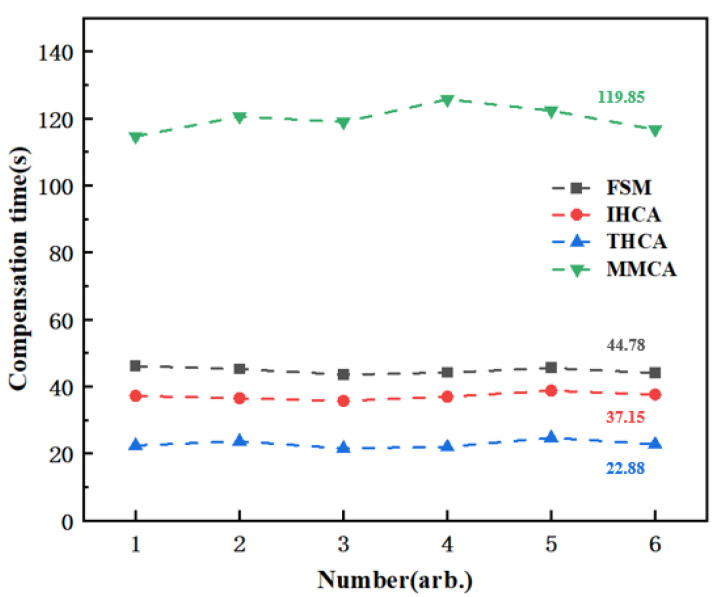
Compensation speeds of four magnetic compensation methods: FSM-IOMCA (44.78 s), IHCA (37.15 s), THCA (22.88 s), and MMCA (119.85 s).

As shown in Figure 12, we compared the compensation speeds of four magnetic compensation schemes: MMCA, THCA, IHCA, and FSM-IOMCA. The FSM-IOMCA scheme enhanced magnetic compensation accuracy through multiple iterations of optimization. While this iterative process led to a moderate reduction in compensation speed compared to THCA and IHCA, FSM-IOMCA remained significantly faster than the MMCA method. Overall, FSM-IOMCA achieved a better balance between compensation speed and accuracy.

As a consequence of the resonance response continually fluctuating in the vicinity of the zero field, compensation resolutions were primarily constrained by three-direction coils [21]. Given that the minimum resolution of FG was 0.1 mV (corresponding to 2 μA) and the minimum resolution of LIA was 0.01 mV (corresponding to 0.2 μA), three directions of magnetic field compensation resolutions could be obtained through coil constants, resulting in 54 pT, 41 pT, and 8 pT, respectively, which were all above the system’s noise level.

**Figure 13:**
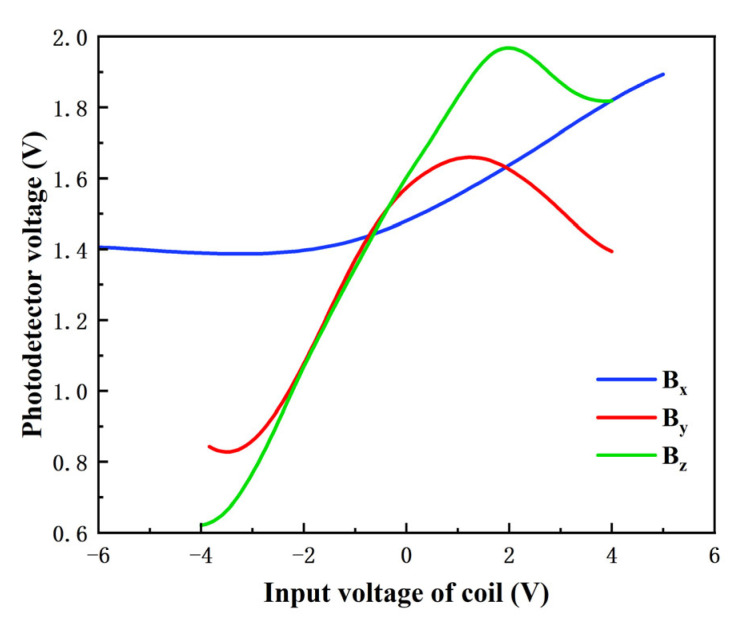
Function generator (FG) scanned x- and y-axes, lock-in amplifier (LIA) scanned z-axis, and a range of magnetic fields recorded photodetector signals *P_PD_*.

To determine the remanent magnetic field in the system, a ± 6 V voltage (corresponding to ±120 mA current) was applied to each axis of the three-axis compensation coil, and the photodetector signals *P_PD_* were recorded accordingly. As shown in Figure 13, when the input voltage to the x-axis coil was approximately −3.168 V (corresponding to −63.36 mA), the scanning curve reached its minimum value. For the y- and z-axes coils, the scanning curves reached their maximum values at approximately 1.228 V (corresponding to 24.56 mA) and 2.020 V (corresponding to 40.4 mA), respectively.

According to the results presented in Section 3, the extreme values of scan curves correspond to the compensation currents required to cancel the remanent magnetic field in each axis. Using the coil constants, the remanent magnetic field components along the x-, y-, and z-axes were calculated to be 1714.52 nT, 506.67 nT, and 1678.22 nT, respectively. These remanent magnetic fields were primarily attributed to interference from the heating system.

Using the FSM-assisted Iterative Optimization Magnetic Compensation Algorithm (FSM-IOMCA) for ten repeated compensations, the compensation voltage of the x-axis was −3.128 ± 0.01 V (corresponding to −62.56 ± 0.2 mA), while the compensation voltages of the y- and z-axes were 1.222 ± 0.005 V (corresponding to 24.44 ± 0.1 mA) and 2 ± 0.005 V (corresponding to 40 ± 0.1 mA), respectively. The three-direction remanent magnetic fields were calculated to be 1692.87 ± 5.4 nT, 504.20 ± 2 nT, and 1661.6 ± 4 nT, respectively. Compared with the above-mentioned remanent magnetic field obtained by scanning, the error rates were 1.26 ± 0.31%, 0.49 ± 0.39%, and 0.99 ± 0.24%. Fluctuations in the magnetic field inside the magnetic shield cylinder could be the source of the inaccuracy. These findings establish the basis for future magnetic field compensation in unshielded situations by demonstrating the precision, stability, and capacity of the FSM-IOMCA system to compensate for strong magnetic fields.

## 7. Conclusions

In this paper, we presented a novel FSM-assisted Iterative Optimization Magnetic Compensation Algorithm (FSM-IOMCA) that significantly advanced the performance of biomagnetic measurement systems. The key innovation lies in the integration of the finite state machine (FSM) model with iterative optimization methods, which demonstrates several major advantages: (1) it decomposes the complex compensation process into well-defined discrete states and transition rules through FSM, substantially improving system stability and operational reliability; (2) the incorporated iterative optimization algorithm enhances compensation accuracy while effectively suppressing crosstalk effects; (3) the sensitivity of the method is improved by 80%, 55%, and 38% compared with the MMCA, THCA, and IHCA methods, respectively; and (4) a pT-level compensation resolution with an error below 1.6% is achieved. The system is built on a single-beam SERF-based magnetometer probe that employs 1 × 2 PMF coupling, which can be further developed into 1 × N PMF coupling, enabling multi-channel biomagnetic measurements to provide enhanced spatial resolution, improved signal-to-noise ratios, and the ability to simultaneously capture multiple physiological signals, leading to more comprehensive and accurate biomagnetic data.

## Figures and Tables

**Table 1:** Expression for the nuclear spin deceleration factor *Q*(*P*).

Nuclear Spin *I*	*Q*(*P*)	*q_lp_*	*q_hp_*
3/2	$\frac{6+2P^{2}}{1+P^{2}}$	6	4
5/2	$\frac{38+52P^{2}+6P^{4}}{3+10P^{2}+3P^{4}}$	38/3	6
7/2	$\frac{22+70P^{2}+34P^{4}+2P^{6}}{1+7P^{2}+7P^{4}+P^{6}}$	22	8

## Data Availability

The data used to support the findings of this study are available from the corresponding author upon request.
